# The Effect of Y Content on Structural and Sorption Properties of A_2_B_7_-Type Phase in the La–Y–Ni–Al–Mn System

**DOI:** 10.3390/molecules28093749

**Published:** 2023-04-27

**Authors:** Emil H. Jensen, Loris Lombardo, Alessandro Girella, Matylda N. Guzik, Andreas Züttel, Chiara Milanese, Pamela Whitfield, Dag Noréus, Sabrina Sartori

**Affiliations:** 1 Department of Technology Systems, University of Oslo, Gunnar Randers vei 19, 2027 Kjeller, Norway; matylda.guzik@its.uio.no (M.N.G.); sabrina.sartori@its.uio.no (S.S.); 2 Institute for Integrated Cell-Material Sciences (iCeMS), Kyoto University, Yoshidahonmachi, Sakyo Ward, Kyoto 606-8317, Japan; lombardo.loris.75a@st.kyoto-u.ac.jp; 3 Department of Chemistry—Physical Chemistry Division, University of Pavia & C.S.G.I., Viale Taramelli 16, I-27100 Pavia, Italy; alessandro.girella@unipv.it (A.G.); chiara.milanese@unipv.it (C.M.); 4 EPFL SB ISIC LMER, EPFL Valais, Rue de I’Industrie 17, CH-1951 Sion, Switzerland; andreas.zuettel@epfl.ch; 5 Excelsus Structural Solutions (Swiss) AG, Park InnovAARE, 5234 Villigen, Switzerland; pamela.whitfield@excelsus2s.com; 6 Department of Materials and Environmental Chemistry, University of Stockholm, Svante Arrhenius Väg 16C, 106 91 Stockholm, Sweden; dag.noreus@mmk.su.se

**Keywords:** hydrogen, hydrogen storage, metal hydrides, compressed gas, intermetallics, A_2_B_7_, La–Y–Ni

## Abstract

Metal hydrides are an interesting group of chemical compounds, able to store hydrogen in a reversible, compact and safe manner. Among them, A_2_B_7_-type intermetallic alloys based on La-Mg-Ni have attracted particular attention due to their high electrochemical hydrogen storage capacity (∼400 mAh/g) and extended cycle life. However, the presence of Mg makes their synthesis via conventional metallurgical routes challenging. Replacing Mg with Y is a viable approach. Herein, we present a systematic study for a series of compounds with a nominal composition of La_2-*x*_Y_*x*_Ni_6.50_Mn_0.33_Al_0.17_, x = 0.33, 0.67, 1.00, 1.33, 1.67, focusing on the relationship between the material structural properties and hydrogen sorption performances. The results show that while the hydrogen-induced phase amorphization occurs in the Y-poor samples (x < 1.00) already during the first hydrogen absorption, a higher Y content helps to maintain the material crystallinity during the hydrogenation cycles and increases its H-storage capacity (1.37 wt.% for x = 1.00 vs. 1.60 wt.% for x = 1.67 at 50 °C). Thermal conductivity experiments on the studied compositions indicate the importance of thermal transfer between powder individual particles and/or a measuring instrument.

## 1. Introduction

Integrating renewables with various energy storage technologies into the electrical grid is a compelling approach for further expansion of the green energy production and utilization [1]. Hydrogen as a fuel and an energy carrier is a part of the sustainable energy revolution, while energy can be stored in a form of liquid or gaseous hydrogen, a considerable amount of energy is lost during the gas compression and/or liquefaction processes [2,3]. An alternative storage method utilizes metal hydrides, in particular intermetallic alloys, which can reversibly sorb hydrogen gas at moderate temperatures and pressures [3,4], in a safer way than the mentioned, conventional solutions [5].

Various metal hydrides have been tested as solid-state hydrogen storage materials, including: AB-, AB_2_-, AB_3_-, AB_5_-, A_2_B_7_- and A_5_B_19_-type compounds, with the A element (e.g., La, Ce, Li, Y) having high-and the B element (e.g., Ni, Fe, Mn, Al) having low affinity to hydrogen [6,7,8,9,10,11].

Previous studies have shown that by performing multiple substitutions of both the A element, and the B elements, the stability of the structure during cycling and storage capacities are affected [12,13,14,15]. It has been found that by substituting Mg for La in rare-earth (RE) superlattice based A_2_B_7_-type metal hydrides, it is possible to achieve a higher storage capacity (compared to commercial AB_5_-type alloys), a lower self-discharge if used in batteries and improved cycle stability by preventing the occurrence of hydrogen-induced amorphization (HIA), when compared to pure La_2_Ni_7_ [12,16,17,18,19,20,21,22].

However, an issue related to using RE-Mg-Ni based materials is due to the high vapour pressure of Mg, making compositional control difficult while also being an explosion hazard [12]. Further, RE-Mg-Ni based materials still suffers from poor cycle stability.

To solve some of these issues, new manufacturing techniques such as smelting protection with helium gas [20,23], sintering techniques [24,25], ball milling [26] or other processing techniques [23,27] have been proposed. These are however either very expensive and/or are highly complicated processes. Evidence suggest that though substitution with Mg is preferable from an economic standpoint, substitution with Y eliminates the issues of Mg synthesis.

Substitution of the La element for the heavier lanthanides is of limited interest for industrial applications due to the cost of these elements, alongside the increase in weight of the final product. However, using Y instead of Mg is a viable solution, since it is lighter and cheaper than the heavier lanthanides. Baddour-Hadjean et al. [28] found that by combining LaNi_5_ and YNi_2_ in appropriate amounts, a La-Y-Ni based AB_3_-type system was formed. It was found that the LaY_2_Ni_9_ alloy was a PuNi_3_-type compound, similar to that of LaMg_2_Ni_9_, and could absorb a higher hydrogen content than that of LaMg_2_Ni_9_ under identical conditions. However, using LaY_2_Ni_9_ as an electrode, showed an electrochemical capacity of only 265 mAh/g. Further, Belgacem et al. [29] showed that LaY_2_Ni_9_ only maintained 54% capacity after 100 cycles. Other studies showed that multi-substituted materials based on the A_2_B_7_-type in the La-Y-Ni system, show great improvements in performance, outperforming already commercialized AB_5_-type metal hydrides [12,30,31,32].

The A_2_B_7_ intermetallic compounds crystallise either with the hexagonal (*H*-; the Ce_2_Ni_7_ structure type; space group: *P6_3_/mmc*, a high-temperature phase) or rhombohedral (*R*-; the Gd_2_Co_7_ structure type, space group: *R-3m*, a low-temperature phase) symmetry. Both polymorphs belong to the family of superlattice compounds with a composite structure, built up by AB_5_ (the CaCu_5_ structure type) and A_2_B_4_ (AB_2_) subunits (the MgZn_2_ and MgCu_2_ structures types, known as C-14 and C-15 *Laves* phases, in the hexagonal and rhombohedral system, respectively) alternated along *c*-axis, with a 2:1 ratio. Due to this, the A_2_B_7_ phases combine the advantage of the fast activation of AB_5_ phases with the high discharge capacity of AB_2_-type compounds.

Yan et al. [12] showed that the hexagonal LaY_2_Ni_9.7_Mn_0.5_Al_0.3_ could absorb up to 1.48 wt.% of hydrogen, which is close to the reported hydrogen storage capacities of the *H*-A_2_B_7_ phases in the RE-Mg-Ni system (up to 1.57 wt.% of H_2_) [18,33].

Liu et al. [34] and Zhao et al. [35] studied the effects of yttrium substitution in A_2_B_7_-type phases of La-Y-Ni-Mn-Al and found that Y induced the formation of the *H*-A_2_B_7_ compositions. These observations however contradict other studies indicating that the yttrium presence favoured the *R*-A_2_B_7_ phase formation [36,37]. Zhao et al. [35] also reported that the studied La_1-*x*_Y_*x*_Ni_3.25_Mn_0.15_Al_0.10_ (x = 0.00–1.00) intermetallic compounds had a single hydrogen ab/desorption plateau, while Liu [34] reported on the formation of two hydrogen ab/desorption plateaus for a similar sample series, La_3-*x*_Y_*x*_Ni_9.7_Mn_0.5_Al_0.3_ (x = 1, 1.5, 1.75, 2, 2.25, 2.5), which is in line with observations reported for other A_2_B_7_-type materials [12,37,38].

With the aim to clarify inconsistencies on structural and functional properties of A_2_B_7_-type materials in the La-Y-Ni-Al-Mn system, we have conducted a systematic study of the sample series La_2-*x*_Y_*x*_Ni_6.50_Mn_0.33_Al_0.17_ (x = 0.33, 0.67, 1.00, 1.33, 1.67), focusing on the analysis of the material structural/microstructural properties and hydrogen sorption behaviour.

## 2. Results

### 2.1. HR SR-PXD and PND

**Figure 1:**
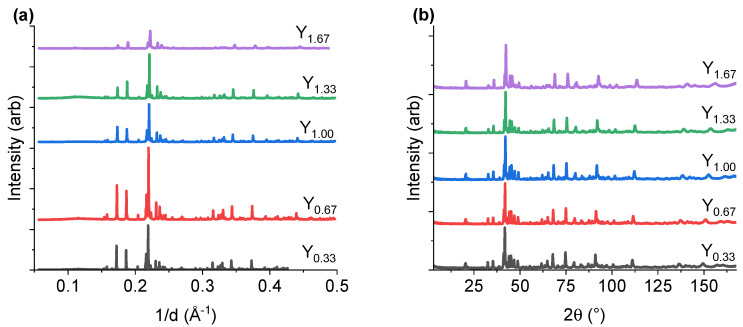
(**a**) HR SR-PXD data (the measurement for Y_0.33_ (black line) was collected with λ = 0.7289 Å, the rest was taken at λ = 0.7291 Å) and (**b**) PND data with λ = 1.54 Å of La_2-*x*_Y_*x*_Ni_6.50_Mn_0.33_Al_0.17_ (x = 0.33, 0.67, 1.00, 1.33, 1.67). The data are given a vertical offset, allowing for easier reading.

**Figure 2:**
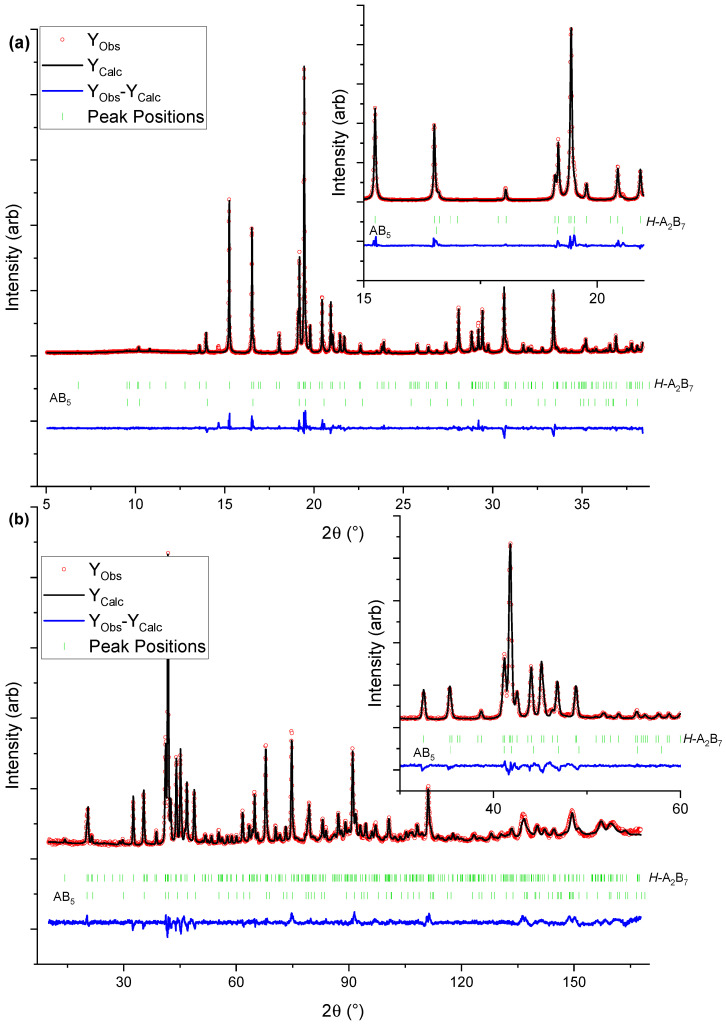
Graphical representation of the Rietveld refinement results for Y_0.33_: (**a**) HR SR-PXD data (λ = 0.7289 Å), (**b**) PND data (λ = 1.5400 Å). The inserts are zoom in of selected ranges.

**Figure 3:**
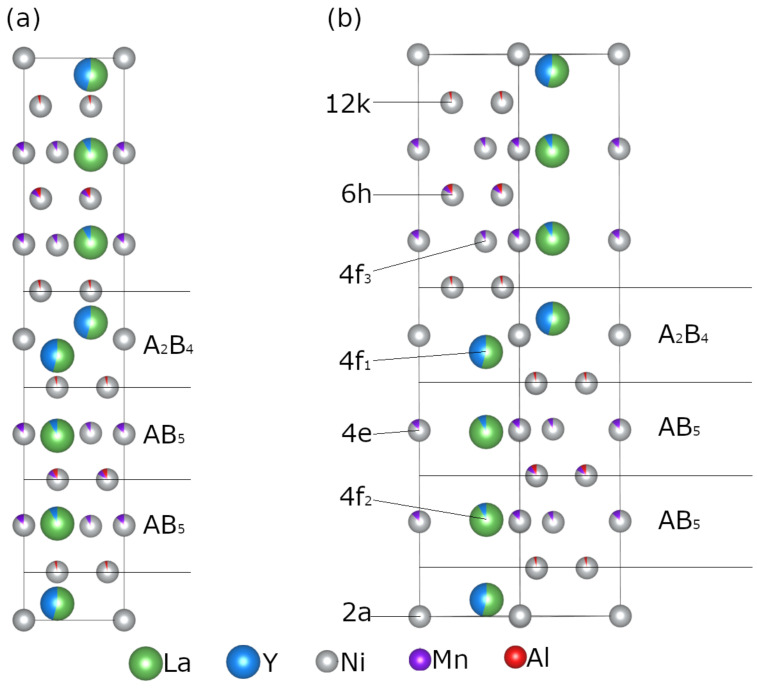
(**a**) Stacking arrangement of the A_2_B_4_ and AB_5_ subunits in the crystal structure of the hexagonal A_2_B_7_-type structure; (**b**) crystallographic sites in the *H*-A_2_B_7_ crystal structure with the fractional atomic occupancies (green: La, blue: Y. grey: Ni, purple: Mn, red: Al atoms) as obtained for the Y_0.67_ sample.

The structural properties of the intermetallic powders were investigated using both HR SR-PXD and PND techniques to obtain sufficient contrast among the system elements, while SR-PXD data helped to differentiate between La and Y, the PND measurements allowed to distinguish the Mn and Ni atoms. The combined simultaneous analysis of both data sets, carried out for each composition, appears essential for the accurate analysis of the material crystal structures (Figure 1, Figure 2 and Figure 3 and Table 1, Table 2, Table 3 and Table 4). The results confirm the formation of *H*-A_2_B_7_ as a main phase in all studied samples. The higher Y content results in the shifting of Bragg peak positions towards higher 2θ angles in the collected diffraction patterns, which indicates the formation of a smaller unit cell, as expected (atomic radius of La = 1.87 Å and Y = 1.79 [39]) and previously reported [34,35]. The substitution of Ni by Al or Mn atoms (atomic radius of Ni = 1.26 Å, Al = 1.41 Å, Mn = 1.32 Å [39]), were expected to influence the average unit cell size, as also reported by other studies [30,38].

The Rietveld refinement results indicate the formation of a single hexagonal Ce_2_Ni_7_-type phase only in Y_0.67_ and Y_1.00_. In all other samples, additional secondary phases are present (Table 1). Y_0.33_ consists of *H*-A_2_B_7_ (95 wt.%) and the minor AB_5_-type (5.3 wt.%) phase. The concentration of the major *H*-A_2_B_7_ increases to 100 wt.% in Y_0.67_ and Y_1.00_ but again decreases to 89 wt.% and 53 wt.% for Y_1.33_ and Y_1.67_, respectively. In the samples with the higher Y concentration (x ≥ 1.33), the formation of the *R*-A_2_B_7_ and *R*-AB_3_ phases has also been observed, which confirms but also contradicts some of the previously reported results. Liu et al. [34] (La_3-*x*_Y_*x*_Ni_9.7_Mn_0.5_Al_0.3_ (x = 1, 1.5, 1.75, 2, 2.25, 2.5)) claimed that the increased Y concentration stabilised the formation of *H*-A_2_B_7_, whereas Zhao et al. [35] (La_1-*x*_Y_*x*_Ni_3.25_Mn_0.15_0Al_0.10_ (x = 0.00–1.00)) observed the presence of the *H*-A_2_B_7_ phase only up to x ≥ 0.85, and the *R*-AB_3_ phase occurrence for higher Y content. The findings presented herein confirm that the Y presence induces the formation *H*-A_2_B_7_ in the entire sample series, but the higher Y concentration (x ≥ 1.33) leads additionally to crystallisation of the *R*-A_2_B_7_ and *R*-AB_3_ phases. Our observations are in line with earlier reports, suggesting that A_2_B_7_ compositions with a higher concentration of smaller A elements, e.g., Y, prefer to take rhombohedral symmetry instead of hexagonal one [40,41], which is in line with the higher abundance of the *R*-A_2_B_7_ phase in Y_1.33_ and Y_1.67_.

The refined lattice parameters of the major *H*-A_2_B_7_ phase decrease with the increasing Y content (***a*** = ***b*** = 5.0740 and 5.0004 Å for Y_0.33_ and Y_1.67_, respectively; ***c*** = 24.643 and 24.322 Å for Y_0.33_ and Y_1.67_, respectively), resulting in smaller subunit (ΔV_A_2_B_4__ = 6.4% and ΔV_AB_5__ = 2.9%) and unit cell volumes (ΔV = 4.2%) as seen in Table 1. (The subunit volumes are calculated using $|\frac{V_{cell}}{c}\cdot \left(\left(z_{1}-z_{2}\right)\cdot c\right|$ where $V_{cell}$ is the unit cell volume, ***c*** is the lattice parameter, and $z_{1}$ and $z_{2}$ are z value coordinates between 0 and 1 of the atoms at the edge of the subunit).

To help determine the quality of the fitted parameters, the *R_wp_* and *R_Bragg_* are shown in Table 2 and Table 3, respectively. As seen for *R_wp_*, the values might seem high. Still, one must remember that when dealing with higher quality datasets, these may, in turn, provide higher *R_wp_* values due to imperfections not considered in the data refining step. On the other hand, the *R_Bragg_* values are generally small, indicating that the chosen model is satisfactory.

As shown in Table 4, the Y occupancy factor at the *4f_1_* and *4f_2_* sites in *H*-A_2_B_7_ increases from 0.14 to 0.70, and from 0.09 to 0.26 for Y_0.33_ and Y_1.00_, respectively. With the higher Y content, the element concentration at the *4f_1_* and *4f_2_* sites rise further to reach eventually 93 and 84%, respectively, in Y_1.67_. This suggests that while initially, Y occupy the A_2_B_4_ subunits, once filled up (x ≥ 1), it prefers entering the AB_5_ subunits. Such behaviour has been previously reported and was related to a lower coordination number of 16 at the *4f_1_* site when compared with coordination number 20 at the *4f_2_* site [36]. The higher Y concentration in the samples also shortens the *4f_1_*-*4f_1_* distance in the neighbouring unit cells from 3.282(3) to 3.170(7) Å for Y_0.33_ and Y_1.67_, respectively.

The Rietveld refinement results indicate that Mn atoms are present at the *4e* and *6h* sites in all studied compositions. The obtained fractional occupancies are low but larger than the estimated standard deviation values. In Y_0.33_, Y_0.67_ and Y_1.00_, Mn appears additionally at the *4f_3_* site. This site depletion observed in the Y-rich compositions, correlates with a subtle increase of the Mn atom occupancies at *4e* and *6h*, similar to the result reported by Deng et al. [31] for Y_0.75_La_0.25_Ni_3.2_Mn_0.3_ with *H*-A_2_B_7_ crystal structure.

In Y_0.33_, Y_0.67_ and Y_1.00_, the Al atoms are distributed over the *6h* and *12k* sites, with the latter being less occupied. This is in line with the earlier report by Wang et al. [38]. They found that for *H*-A_2_B_7_ in the LaY_1.9_Ni_10.2-*x*_Al_*x*_Mn_0.5_ (x = 0, 0.2, 0.4, 0.6) system the presence of Al was only detected at the *6h* site, between two AB_5_ subunits. Similar results were also observed for *H*-A_2_B_7_ formed in La_0.77_Mg_0.23_Ni_3.41_Al_0.09_ and Nd_0.9_Mg_0.1_Ni_3.3_Al_0.2_ [21,42]. In Y_1.33_ and Y_1.67_, the *6h* site is no longer populated by Al, but at the same time, a slight increase of aluminium concentration at the *12k* site is observed. These findings, compared with the changes in population of the *4f_1_* and *4f_2_* sites, may indicate that the higher amount of Y atoms in *H*-A_2_B_7_, and its increasing concentration at the *4f_2_* site, have an effect on (correlates with) the B atom distribution over the available crystallographic sites in this crystal structure, however the nature of interatomic relationships must be further investigated.

### 2.2. In Situ XRD

**Figure 4:**
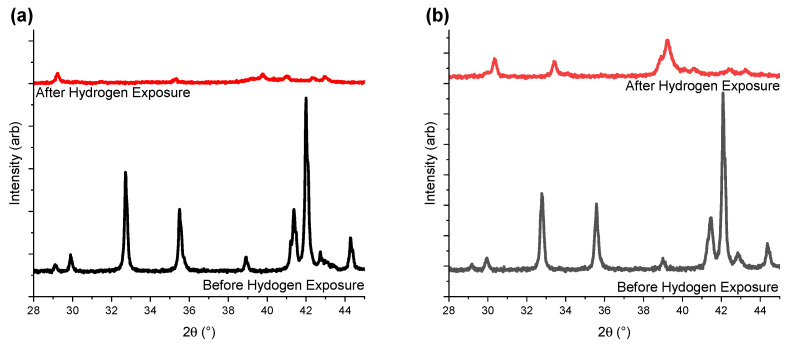
In situ PXD data (λ = 1.5406 Å) collected for (**a**) Y_0.67_ (30 °C, 8 bar of H_2_) and (**b**) Y_1.00_ (30 °C, 20 bar of H_2_) before and after hydrogenation. The black lines correspond to the pristine intermetallic compounds, while the red ones represent the hydrogenated materials. Data for the remaining samples can be found in Supplementary Information (Figure S1). The data are given a vertical offset, allowing for easier reading.

In situ PXD data were collected to determine: (1) the experimental conditions required for the hydrogenation of the studied compounds, and (2) the phase compositions of the hydrogenated materials. After initial activation of sample Y_0.33_ and Y_0.67_, various hydrogen pressures and temperatures were applied, within the ranges reported previously in literature [12]. Regardless of the tested experimental conditions, both materials became amorphous upon hydrogenation. Figure 4a shows the results obtained for Y_0.67_ at 30 °C and under 8 bar of H_2_ (red line). Due to the amorphous nature of the H_2_ exposed Y_0.33_ and Y_0.67_, the samples were excluded from further studies. The Y-rich samples (x ≥ 1) were successfully hydrogenated at 20 bar of H_2_ and 30 °C, with well-preserved crystallinity of the formed metal hydride phases (red plots in Figure 4b and Figure S1 in the Supplementary Information).

The previously reported investigation also indicated the occurrence of HIA for similar intermetallics (e.g., La_1-*x*_Y_*x*_Ni_3.25_Mn_0.15_Al_0.10_) at lower Y concentration (x < 0.5) [35]. Aoki and Masumoto [43] related the probability of the HIA occurrence with the ratio of the Goldschmidt radii between the A and B atoms ($R_{A}/R_{B}$). Based on the empirical data, if the ratio was below 1.37 ($R_{A}/R_{B}$ < 1.37) for the A_2_B_4_ subunit, then HIA was effectively prevented, whereas if above amorphization was expected to happen. The calculated Goldschmidt radii ratio in our samples decreased from 1.48 for Y_0.33_ to 1.43 for Y_1.67_, being higher than the limit found by Aoki and Masumoto. A similar result was reported for La_1-*x*_Y_*x*_Ni_3.25_Mn_0.15_Al_0.1_ in [35] where a ratio of 1.453 was reported, with the *4f_1_* site being completely filled with Y. The difference in the calculated ratios, was expected to be due to different atomic sizes being used, with the source not being provided in [35]. As such, all samples were expected to get amorphous.

It has also been found that the hydrogenated samples reported in this paper spontaneously release hydrogen, when stored in a sealed stainless-steel vial stored in a glovebox under ambient argon atmosphere (Figure S2 in Supplementary Information).

### 2.3. SEM and EDX

**Figure 5:**
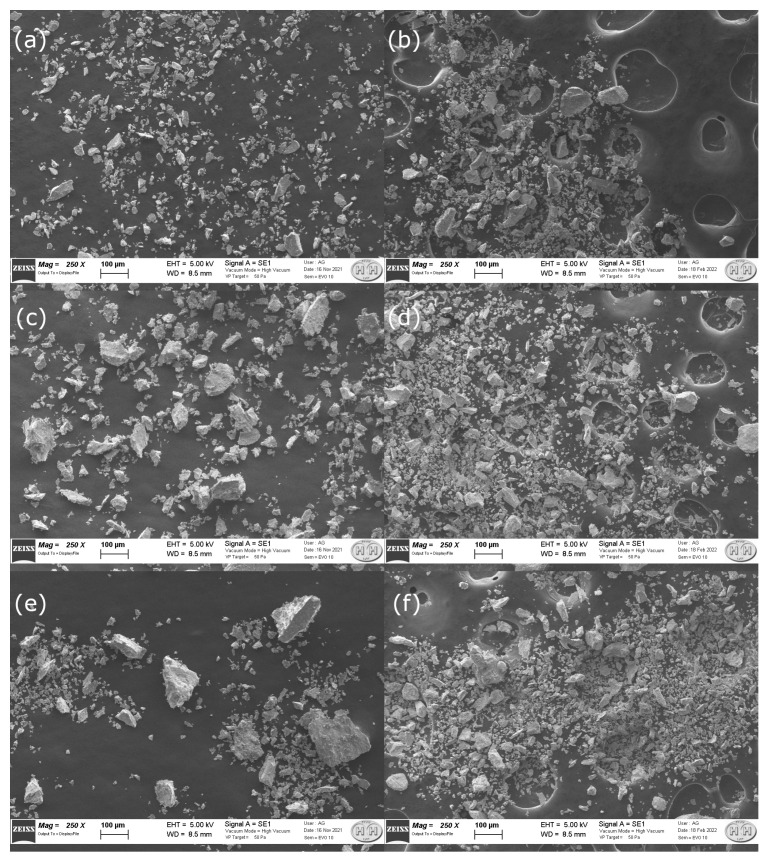
SEM micrographs collected at 250× for: Y_1.00_, Y_1.33_ and Y_1.67_ before (**a**, **c** and **e**, respectively) and after (**b**, **d** and **f**, respectively) hydrogen exposure. Additional micrographs obtained at higher magnification (25,000×) can be found in Supplementary Information (Figure S3).

Investigations with SEM-EDX were carried out for Y_1.00_, Y_1.33_ and Y_1.67_ to: (1) map out the chemical composition of the intermetallics, and (2) to study the effect of the Y concentration on the particle size distribution in the pristine intermetallics and hydrogenated samples. The difference in holes in the carbon tape has no influence to the analysis. These are caused by the quality and type of the carbon tape used. The differences in coverage can be due to three factors. (1) The samples were produced three months apart manually (no reproducible results). (2) The finer material obtained after hydrogenation formed a blanket that was stuck to the carbon tape. (3) Since the micrographs were taken three months apart, the stickiness of the carbon tape could have changed (different roll). Before hydrogen exposure, the Y_1.00_ material (Figure 5a) revealed the most homogeneous particle size distribution as compared to other samples. In contrary, the Y_1.67_ powders (Figure 5e) were characterized by the largest spread of the particle sizes. A zoom onto the particle surface at 25,000× before and after exposure to hydrogen can be seen in Figure S3 in the Supplementary Information. After hydrogenation, smaller particles with a comparable size distribution were observed in all studied compositions (Figure 5b,d,f). Although not directly comparable, Liu et al. [34] found that when the Y concentration increased in La_3-*x*_Y_*x*_Ni_9.7_Mn_0.5_Al_0.3_ (x = 1, 1.5, 1.75, 2, 2.25, 2.5) from x = 1 to x = 1.5, the average particle size increased. Increasing the Y amount further, lowered the average particle size.

EDX investigations (Table 5, Supplementary Information Figure S4) consisted of two sets of data acquired for Y_1.00_, Y_1.33_ and Y_1.67_; one focusing on a small region of a seemingly flat particle, with the second covering a larger area (referred to as “overview” in Table 5). A general trend presented in the EDX data confirmed the lower Ni content than in the sample nominal compositions when looking at single particles. Still, when considering at the overview, Ni concentrations were closer to the nominal values. When comparing the nominal composition, the obtained (average) EDX compositions and the refined compositions, it was still observed that the amount of Ni detected in the EDX data was smaller than for the pristine and refined data (Table 6). Hao et al. [44] found that when annealing samples of La_0.33_Y_0.67_Ni_3.25_Mn_0.15_Al_0.1_, that the composition in the surface change. It is thus believed this is the reason for the lower Ni contant on the surface, and that it instead is located in the bulk. Further, since the samples are not perfectly flat, this can also influence the final results, altering the ratio of different elements detected.

### 2.4. PCT

**Figure 6:**
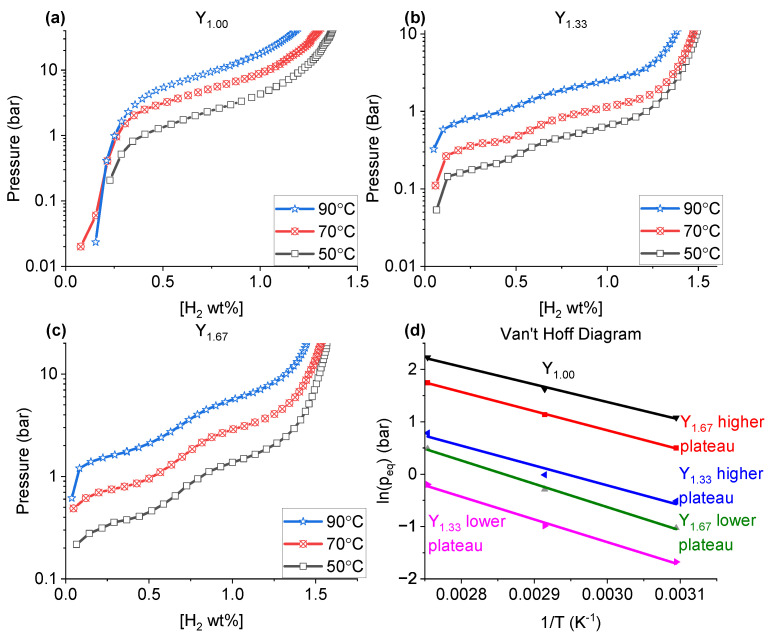
The PCT diagrams for (**a**) Y_1.00_, (**b**) Y_1.33_, (**c**) Y_1.67_ collected at 50, 70 and 90 °C; (**d**) the corresponding Van’t Hoff plots.

The PCT measurements (Figure 6) were performed for Y_1.00_, Y_1.33_ and Y_1.67_ to investigate in detail, the material hydrogen absorption behaviours. The corresponding Van’t Hoff plots are shown in Figure 6d. The values of the enthalpy and entropy of the hydride formation in the studied samples, along with the expected plateau pressures at 30 °C, are listed in Table 7.

No leaks were detected during the pre-hydrogen exposure setup. For each point of the PCT a step time of 1.5 h has been chosen in order to obtain a PCT measurement with satisfactory accuracy. As an example, Figure S5 of the supplementary information shows a representative kinetics curve obtained for one point during the PCT measurements.

As highlighted in the paper by Rudman [45], great care needs to be taken when measuring the PCT characteristics of a material. A very high ΔP (40 bars) was chosen during activation, and hydrogen was completely absorbed within minutes. During the PCT measurements, a ΔP of 2 bars was set for each point, with the sample experiencing a ΔP of approximately 1 bar due to the reservoir and sample holder volumes described in the experimental section. Here, most hydrogen had been absorbed within minutes, with the absorption being greatly slowed down after 30 min of hydrogen exposure.

While the PCT plot for Y_1.00_ (Figure 6a) revealed only one clearly visible plateau pressure, at all studied temperatures, the data for Y_1.33_ and Y_1.67_ (Figure 6b,c) displayed two hydrogen absorption regions. Furthermore, the single plateau of Y_1.00_ occurs at a higher hydrogen pressure value (2.9 bar of H_2_ at 50 °C) than any of the highest plateaus observed for Y_1.33_ and Y_1.67_ (0.6 and 1.6 bar of H_2_ at 50 °C for Y_1.33_ and Y_1.67_, respectively). Previous studies showed that for La_2_Ni_7_-based materials, either single [46] or multiple [37] plateau pressures can be expected for both single phase (*H*-A_2_B_7_ or *R*-A_2_B_7_) or combined phase materials. It has been discussed that if more than one plateaus were present, it was likely due to: (a) the existence of multiple hydrogen-active (absorbing) phases in the sample [47], and/or (b) the hydrogen absorption at different pressures by various structural subunits [37]. The PCT data presented in Figure 6 differ from those reported in literature for similar compositions, both in the number of plateaus and, in the case of Y_1.00_, the equilibrium pressures [34,35,37]. Single plateaus in A_2_B_7_-type compounds can be obtained by adjusting the subunit volumes [37].

As mentioned earlier, there can be two potential reasons for the appearance of the two plateaus as observed in Y_1.33_: The appearance of multiple phases [47], or subunits absorbing at different pressures [37]. Since both samples contained multiple phases, it could be assumed that this was the reason for the multiple plateaus. However, for La_2-*x*_Y_*x*_Ni_7_ Zhang et al. [37] argued that the appearance of the multiple plateaus were not due to the *H*-A_2_B_7_ or *R*-A_2_B_7_ phases appearing at the same time, but rather the different subunits absorbing at different pressures due to a difference in volume size.

For Y_1.67_ (Table 1), a *R*-AB_3_ phase was present in addition to the *H*-A_2_B_7_ and *R*-A_2_B_7_-type phases. Earlier studies have found that the equilibrium pressures of *R*-AB_3_ were expected to be similar to that of *H*-A_2_B_7_ due to the similarities in their superstacking structures [48,49]. As such, it was considered that the appearance of two plateaus in Y_1.33_ and Y_1.67_ was not due to the appearance of different phases, but due to differences in the subunit volumes in these phases.

By increasing the ratio Y/La our measurements showed a higher gas storage capacity (1.37 wt.% for Y_1.00_ vs. 1.60 wt.% for Y_1.67_, both at 50 C (Table 8)). This was partly attributed to the lower weight of Y compared to La, though the large increase in capacity from Y_1.00_ (1.37 wt.% at 50 °C) to Y_1.33_ (1.57 wt.% at 50 °C) cannot be explained by the sole relative weight difference between La and Y.

When performing PCT at various temperatures, for each sample a loss of storage capacity was observed as the temperature increase (Figure 6 and Table 8), with a more marked change from 70 °C to 90 °C (between 0.05 and 0.10 wt.% difference) than from 50 °C to 70 °C (between 0.02 and 0.05 wt.% difference). Furthermore, the major changes were observed in Y_1.00_, compared to the changes for Y_1.33_ and Y_1.67_.

According to various findings, the plateau pressure values are affected by either a local chemical (binding) environment between the hydrogen atoms and the metallic atoms in the crystal structure (both A and B elements) [50], and/or differences in unit cell size [51]. According to Khatamian and Manchester [52], the formation enthalpy of YH_2_ is −219.6 kJ/mol(H_2_) close to other findings [53], whereas that of LaH_2_ is −208 kJ/mol(H_2_) [50,54]. This difference indicates that a higher La-content in the sample should result in higher plateau pressure values. In our case, as indicated by the values obtained for Y_1.33_ and Y_1.67_ (Figure 6b,c) it was observed that with increasing Y content the plateaus increased. It is important to note that the plateau pressure were higher for Y_1.00_ (Figure 6a), when compared to Y_1.33_ and Y_1.67_. This goes against the expected trend, seeing as others have shown for A_2_B_7_-type materials based on La-Y-Ni the plateau increased the more Y was present, due to a decrease in the average unit cell size [34,35,37]. Further, Zhang et al. [37] showed that in case the two plateaus were due to absorption in the different subunits, the lower plateau pressure can be related to the volume of the A_2_B_4_ subunit, in that if the subunit volume decrease, the lower plateau pressure increase. The higher plateau was more complex and requires a more thorough inspection, since some substitutions lead to plateau pressure stabilization, even with changes to the subunit volumes [37].

The measured hydrogenation pressures for Y_1.00_ absorbed from 2.9 bar at 50 °C to 9.3 bar at 90 °C, for Y_1.33_ the lower plateau pressure absorb from 0.2 bar at 50 °C to 0.8 bar at 90 °C, the higher pressure go from 0.6 bar at 50 °C to 2.2 bar at 90 °C. Y_1.67_ displayed a lower plateau absorbing from 0.4 bar at 50 °C to 1.6 bar at 90 °C and a higher plateau of 1.6 bar at 50 °C to 5.7 bar at 90 °C. From these measurements the enthalpy and entropy of formation, using a Van’t Hoff plot as shown in Figure 6d. From these, the pressure plateaus at 30 °C were calculated (Table 7).

Liu et al. [34] found that for La_3-*x*_Y_*x*_Ni_9.7_Mn_0.5_Al_0.3_ (x = 1, 1.5, 1.75, 2, 2.25, 2.5) all samples had two plateaus (found via electrochemical measurements). For a composition similar to Y_1.00_ (La_1.5_Y_1.5_Ni_9.7_Mn_0.5_Al_0.3_) the plateaus were at roughly 0.02 bar and 0.07 bar, for La_1.0_Y_2.0_Ni_9.7_Mn_0.5_Al_0.3_ (similar to Y_1.33_) the plateaus were at 0.06 bar and 0.2 bar. Lastly for La_0.5_Y_2.5_Ni_9.7_Mn_0.5_Al_0.3_ (similar to Y_1.67_) the plateaus were at 0.07 bar and 0.3 bar.

Zhao et al. [35] found that La_1-*x*_Y_*x*_Ni_3.25_Mn_0.15_Al_0.10_ (x = 0.00–1.00) showed both single and multiple plateau behaviour. When looking at the compositions La_0.5_Y_0.5_Ni_3.25_Mn_0.15_Al_0.10_, La_0.33_Y_0.67_Ni_3.25_Mn_0.15_Al_0.10_ and La_0.12_Y_0.85_Ni_3.25_Mn_0.15_Al_0.10_ (comparable to Y_1.00_, Y_1.33_ and Y_1.67_, respectively) only single plateaus were observed at 0.26 bar, 0.54 bar and 0.94 bar, respectively.

When comparing all of these literature plateau pressure values with the plateau pressures calculated at 30 °C, it was observed that for Y_1.00_ the calculated plateau was higher than reported values in the literature for both single and multiple plateaus. When comparing the calculated plateau pressures of Y_1.33_ and Y_1.67_ to the plateau pressures found by Liu et al. [34] it was observed that all calculated plateau pressures reported here were higher, than the electrochemically measured plateaus reported by Liu et al. By comparing the calculated plateau pressures to those obtained by Zhao et al. [35], it was observed that the single plateaus reported by Zhao et al. have higher plateau pressures, than both of the calculated plateau pressures.

As described, there are no clear trends in the literature regarding this compound, both regarding plateau pressures, but also the number of plateaus. The reported data correspond to this in that no clear trends were found, showing that the picture is more complex, and great care needs to be applied to all known aspects that can affect the hydrogenation properties.

**Figure 7:**
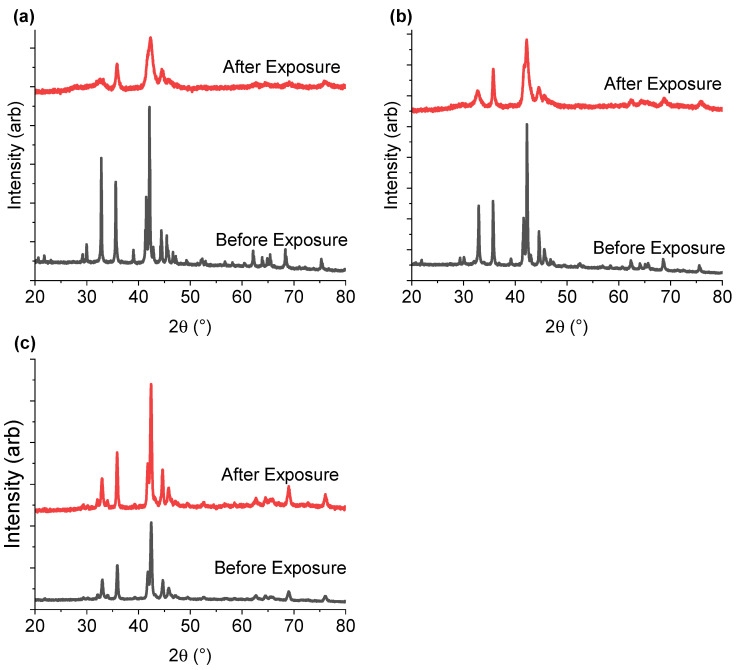
PXD data for (**a**) Y_1.00_, (**b**) Y_1.33_, and (**c**) Y_1.67_ collected before and after PCT experiments. Red plots correspond to the intermetallic compound after four absorption/desorption cycles (one activation cycle, and three PCT cycles). The black lines correspond to the pristine intermetallic compound. The data are given a vertical offset, allowing for easier reading.

Figure 7 shows the PXD data collected for the three samples before and after PCT experiments, with sample completely desorbed (here called after exposure). The sample with the lowest yttrium concentration (Y_1.00_) become less crystalline after exposure to hydrogen gas as compared to Y-rich compositions. This suggests that higher Y content helps to retain material crystallinity during hydrogen ab/desorption.

Fang et al. [55] found that if the volumes of the A_2_B_4_ and AB_5_ subunits in a variety of AB_3_-type intermetallics (PrNi_3_, NdNi_3_, SmNi_2.67_Mn_0.33_, SmNi_3_, Sm_0.9_Mg_0.1_Ni_3_ and Nd_0.33_Er_0.67_Ni_3_) were smaller than 89.2 Å^3^ and 88.3 Å^3^, respectively, then the material could release all of the absorbed hydrogen. When above these limits, the hydrogen could not be completely desorbed from their respective subunits. The critical volume limits were further confirmed by Zhang et al. [37], who studied a variety of La_*x*_A_2-*x*_Ni_7_, (A = Gd, Sm, Y, Mg). Samples of La_*x*_Y_2-*x*_Ni_7_ with x between 0 and 0.8, had subunit volumes below these critical values mentioned above, and showed the best cycling behaviour.

Among the three hydrogenated samples presented here, only the A_2_B_4_ and AB_5_ subunits of the Y_1.67_ intermetallic were below the critical values of 89.2 Å^3^ and 88.3 Å^3^ (Table 1). When analysing the XRD data before and after hydrogenation, Y_1.67_ was also the sample with the best-preserved crystallinity after sample activation and three PCT hydrogenation cycles (Figure 7). Thus, the loss in crystallinity and lower capacity for samples with lower Y content may be due to partial hydrogen release. For Y_1.00_ and Y_1.33_, while the volumes of the A_2_B_4_ subunits exceeded the critical values, sizes of the AB_5_ subunits were rather borderline values, with Y_1.33_ being just above the limit. This may explain the appearance of the secondary plateau in the case of Y_1.33_, as well as the better crystallinity and hydrogen release compared to Y_1.00_. In the case of Y_1.67_ the volume values were below the critical limit for both subunits and confirmed the link with better crystallinity compared to the other samples, and the most well-defined plateau regions. Further investigations are needed to confirm this hypothesis.

### 2.5. Thermal Conductance

**Figure 8:**
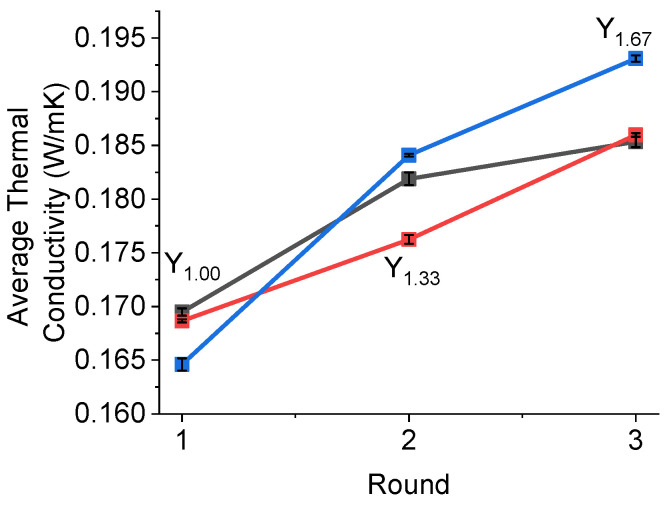
The average thermal conductivity over three rounds (one round was five measurements). The Y-axis was the average value collected in each round.

Thermal conductivity measurements were performed for the intermetallic powders of Y_1.00_, Y_1.33_ and Y_1.67_ (Figure 8). The measurements were conducted over three rounds, with each round having five measurements. Between rounds, the material was moved around, and re-compacted.

The thermal conductivity of hydrogen storage materials is often an overlooked parameter. For intermetallic compounds, the typical value is low and varies between 0.1 and 1 W/mK. While at lab-scale, the limiting factor of hydrogen absorption/desorption often is related to the intrinsic kinetics of hydrogen absorption of the samples [56], for large batches, the hydrogen uptake and release are dependent on the thermal management of the hydrogen storage systems and thermal conductivity of metal hydrides [56].

As can be observed in Figure 8, the data obtained in this study do not suggest any correlation with the Y content or the sample phase compositions. However, while stirring and re-compacting the powders between measurements, higher values of thermal conductivity were achieved, which may indicate that the contact between particles was essential for enhancing the thermal properties of the samples. It was very likely that the higher values of thermal conductivity for Y_1.67_ were due to the greater particle size distribution in this sample, as observed by SEM (Figure 5).

## 3. Experimental Tools and Methods

The series of samples with the following nominal compositions:

La_2-*x*_Y_*x*_Ni_6.50_Mn_0.33_Al_0.17_, x = 0.33, 0.67, 1.00, 1.33, 1.67, hereafter referred to as Y_0.33_, Y_0.67_, Y_1.00_, Y_1.33_ and Y_1.67_, respectively, were prepared according to the synthesis method reported in [12].

The intermetallic powders were studied by high-resolution synchrotron radiation powder X-ray diffraction (HR SR-PXD) at the MCX beamline (Elettra, Basovizza, Italy) [57] (for Y_0.33_: λ = 0.7289 Å; for all remaining compositions: λ = 0.7291 Å; 2θ range: 5–38°, step size: 0.008°2θ). During the measurements, powders were sealed in boron-silica capillaries (d = 0.3 mm) and rotated. The powder neutron diffraction (PND) experiments were carried out with a high resolution diffractometer BT-1 at the National Institute of Standards and Technology (NIST, Gaithersburg, MD, USA) (λ = 1.5400 Å; 2θ range: 3–167°; step size: 0.05°2θ, the Cu(311) monochromator). The powders were measured in a 5 mL vanadium can, sealed with an indium wire.

The hydrogen sorption behaviour of the studied compositions was investigated by in situ powder X-ray diffraction carried out with a laboratory Bruker D8 Advance diffractometer, equipped with an in-house built set-up for hydrogen gas pressurisation and thermal sample heating [58]. The data were collected with Cu Kα_1_ (λ = 1.5406 Å; 2θ range: 20–95°; step size: 0.0176°2θ). The powders, sealed in a beryllium sample holder, were first heated up to 70 °C for 1 h, under dynamic vacuum, and subsequently exposed to static hydrogen pressure in the range of 8–20 bar. The hydrogenated materials were subsequently cooled down to temperatures in the range of 28–30 °C.

The HR SR-PXD and PND data collected for the intermetallic compounds were jointly analysed by Rietveld refinements using the Fullprof Suite program [59]. The diffraction profiles were modelled using the pseudo-Voigt peak shape function with the background being defined by interpolation between manually chosen points. For the final refinement cycles the following parameters were allowed to vary: the scale factors of indexed phases, lattice parameters of indexed phases, up to six profile parameters (U, V, W, mixing factor and two asymmetry parameters) and overall or individual displacement parameters.

To model the hexagonal phase, the La_2_Ni_7_ crystal structure was used as a prototype. Based on the previously reported data, it was assumed that Y could substitute only La atoms, while Mn and/or Al could replace exclusively Ni. Based on the structure prototype the *4f_1_*, *4f_2_* crystallographic sites were assumed to be filled with A elements while *4e*, *4f_3_*, *6h* and *12k* were assumed to be completely filled with B elements. The *2a* site was assumed to be completely filled with Ni and not refined [21,31,38]. During the refinement cycles, the occupancy of the *4f_1_* and *4f_2_* sites were shared between La and Y, and constrained to 100% for each site. Initially, Ni, Mn and Al atoms were evenly distributed over the *4e*, *4f_3_*, *6h* and *12k* sites according to the nominal compositions. However, to correctly determine the multiple (three atoms) atom occupancy at these sites, their distribution in the crystal structure model was constrained as described in [60]. In the final refinement cycle, Ni was distributed over all five sites, with Mn atoms partly present at the *4e*, *4f_3_* and *6h* sites, and Al atoms partially occupying *6h* and *12k*.

Pressure–Composition–Temperature (PCT) measurements were performed with a Hy-Energy PCTPro-2000 Sieverts apparatus. In each measurement, 3 g of the intermetallic samples were placed into a stainless-steel sample holder with a calibrated volume between 12.5 and 13.0 mL. The sample holder was heated using an external thermal couple inserted into the sample holder. The material was first activated under dynamic vacuum at 70 °C for 1 h, and then exposed to hydrogen gas at 40 bar. Subsequently, the hydrogen was removed from the sample, keeping it under dynamic vacuum at 390 °C overnight. Following the activation, the samples were cooled down to 50, 70 and 90 °C, and the PCT hydrogen absorption data collected (ΔP = 2 bar; no steady state criteria was used, instead a step time of 1.5 h for each point was decided on, ensuring each measured point was as close to equilibrium as possible; maximum applied P = 50 bar; V_reservoir_ = 11.530 cm^3^; V_sample holder_ = 12.430–13.298 cm^3^ depending on temperature and sample loading). Before the samples were exposed to hydrogen, the system used He gas to perform an automatic leak test. Prior to and after the three PCT experiments reported, sample phase compositions were investigated with a Bruker D2 PHASER (λ = 1.5406 Å; 2θ range: 20–80°, step size: 0.02°2θ).

The intermetallic and desorbed hydride powders were also investigated by scanning electron microscopy (SEM) and energy dispersive X-ray spectroscopy (EDX) with a Zeiss EVO MA10 microscope.

The thermal conductivity of the intermetallic powders was determined using the modified transient plane source (MTPS) with a C-Therm Trident system.

## 4. Conclusions

The structural and hydrogen storage properties of La_2-*x*_Y_*x*_Ni_6.5_Mn_0.33_Al_0.17_ sample series have been investigated. It was found that Y initially induced the formation of a *H*-A_2_B_7_-type structure, and that higher Y amounts induced the *R*-A_2_B_7_-type and *R*-AB_3_-type structures.

The Rietveld refinement showed that of the two sites (*4f_1_* and *4f_1_*) where La and Y are located, Y initially prefered to enter into the site of the A_2_B_4_ subunit (*4f_1_* site), with a small amount entering the AB_5_ subunit. Instead, when x < 1.00, it was seen that Y primarily entered the AB_5_ subunit, similar to the behaviour of single substituted samples. Further, it was found that Al resided at the *6h* and *12k* sites, while Mn resided at the *4f_3_*, *4e* and *6h* sites at lower Y amounts. When x < 1.00, and Y started entering the AB_5_ subunit, Al was only located on the *12k*, while Mn was only located on the *4e* and *6h* sites. This indicates that the presence of the smaller Y atom on the *4f_2_* site, can influence which sites the substituted B atoms are located at.

It was found that if the Y amount was low, the material became amorphous immediately during hydrogen exposure, and at higher Y-contents the crystal structure of the material was preserved. This has been coupled to the subunit volumes, since it was also found that the subunit volumes of the main *H*-A_2_B_7_-type phase decreased with increasing Y content, leading to a better retention of the crystal structure and more defined plateau pressures. Further, by increasing the amount of Y present in the samples, higher hydrogen storage capacities were achieved.

No definitive conclusions could be drawn about the effects of Y on the thermal conductivity nor anti-pulverization ability, and will need further investigation.

## Figures and Tables

**Table 1:** Lattice parameters, phase abundance, A_2_B_4_ and AB_5_ subunits and unit cell volumes for the *H*-A_2_B_7_ phase in the studied intermetallic compounds, as obtained from Rietveld refinements.

Sample	Phase Structure Type(s)	Phase Abundance [wt.%]	Lattice Parameters		Unit Cell Volume (V) [Å^3^]	A_2_B_4_ Subunit Volume (*V_A_2_B_4__*) [Å^3^]	AB_5_ Subunit Volume (*V_AB_5__*) [Å^3^]
			*a* [Å]	*c* [Å]			
Y_0.33_	*H*-A_2_B_7_	95(1)	5.07420(2)	24.643(1)	549.47(4)	94.70(7)	90.02(5)
	AB_5_	5.3(3)	5.0599(8)	4.0873(7)	90.63(3)	-	-
Y_0.67_	*H*-A_2_B_7_	100	5.0606(2)	24.5522(1)	544.52(4)	93.32(5)	89.47(3)
Y_1.00_	*H*-A_2_B_7_	100	5.0424(2)	24.4558(8)	538.51(3)	91.67(5)	88.80(3)
Y_1.33_	*H*-A_2_B_7_	88(1)	5.03295(6)	24.4083(6)	535.44(2)	90.32(5)	88.70(4)
	*R*-A_2_B_7_	11.3(7)	5.0335(3)	36.614(3)	803.28(9)	-	-
Y_1.67_	*H*-A_2_B_7_	53(1)	5.0004(2)	24.322(2)	526.66(4)	88.59(9)	87.37(6)
	*R*-A_2_B_7_	24.0(6)	4.9993(3)	36.480(4)	789.6(1)	-	-
	*R*-AB_3_	23.0(6)	5.0115(2)	24.423(2)	531.21(6)	-	-

**Table 2:** *R_wp_* values reported from the Rietveld refinement.

Sample	Dataset	*R*_*wp*_
Y_0.33_	X-ray	15.9
	Neutron	16.8
Y_0.67_	X-ray	10.6
	Neutron	18.9
Y_1.00_	X-ray	12.6
	Neutron	13.6
Y_1.33_	X-ray	15.6
	Neutron	15.1
Y_1.67_	X-ray	14.9
	Neutron	12.4

**Table 3:** *R_Bragg_* values reported from the Rietveld refinement.

Sample	Dataset	Phase	*R_Bragg_*
Y_0.33_	X-ray	*H*-A_2_B_7_	6.38
		AB_5_	10.2
	Neutron	*H*-A_2_B_7_	8.79
		AB_5_	10.6
Y_0.67_	X-ray	*H*-A_2_B_7_	4.47
	Neutron	*H*-A_2_B_7_	11.6
Y_1.00_	X-ray	*H*-A_2_B_7_	4.65
	Neutron	*H*-A_2_B_7_	7.17
Y_1.33_	X-ray	*H*-A_2_B_7_	9.59
		*R*-A_2_B_7_	16.7
	Neutron	*H*-A_2_B_7_	4.98
		*R*-A_2_B_7_	7.76
Y_1.67_	X-ray	*H*-A_2_B_7_	5.00
		*R*-A_2_B_7_	7.71
		*R*-AB_3_	4.79
	Neutron	*H*-A_2_B_7_	5.22
		*R*-A_2_B_7_	6.12
		*R*-AB_3_	5.08

**Table 4:** Refined sample compositions, fractional occupancies (***n***), displacement factors (**B_iso_**), atomic positions, atomic distance ***4f_1_***-***4f_1_*** in the A_2_B_4_ subunit and goodness-of-fit ($\chi^{2}$) for the *H*-A_2_B_7_ phase in the studied intermetallic compounds, as obtained from Rietveld refinement. All crystallographic sites are assumed to be fully occupied; the remaining fractional occupancies are completed to 100% by either La (A atoms) or Ni (B atoms).

	Y_0.33_	Y_0.67_	Y_1.00_	Y_1.33_	Y_1.67_
**Refined Composition**	La_1.77_Y_0.23_Ni_6.41_ Mn_0.33_Al_0.26_	La_1.45_Y_0.55_Ni_6.48_ Mn_0.34_Al_0.19_	La_1.04_Y_0.96_Ni_6.47_ Mn_0.33_Al_0.20_	La_0.70_Y_1.30_Ni_6.46_ Mn_0.32_Al_0.23_	La_0.23_Y_1.77_Ni_6.50_ Mn_0.34_Al_0.16_
**A_1_** *4f_1_* ***n*** **_Y_**	0.14(2)	0.47(1)	0.70(2)	0.82(4)	0.93(3)
**B_iso_[Å^2^]**	2.9(1)	2.61(6)	2.02(8)	1.84(9)	2.9(2)
**z**	0.5300(1)	0.52947(8)	0.5292(1)	0.5288(2)	0.5269(3)
***4f_1_*** **-** ***4f_1_*** **[Å]**	3.282(3)	3.260(2)	3.243(3)	3.229(4)	3.170(7)
**A_2_** *4f_2_* ***n*** **_Y_**	0.09(2)	0.08(1)	0.26(2)	0.48(2)	0.84(2)
**B_iso_[Å^2^]**	2.34(8)	3.16(5)	2.33(6)	1.23(8)	0.8(1)
**z**	0.6723(1)	0.67187(8)	0.6717(1)	0.6714(1)	0.6705(3)
**B_1_** *2a* **B_iso_[Å^2^]**	1.1(1)	1.79(9)	1.3(1)	0.8(1)	0.7(2)
**B_2_** *4e* **n_Mn_**	0.15(1)	0.14(1)	0.13(1)	0.17(1)	0.16(2)
**B_iso_[Å^2^]**	2.8(1)	2.88(9)	2.0(1)	1.5(1)	0.4(1)
**z**	0.1691(3)	0.1687(2)	0.1680(2)	0.1687(3)	0.1671(4)
**B_3_** *4f_3_* ***n*** **_Mn_**	0.06(1)	0.066(9)	0.054(8)	-	-
**B_iso_[Å^2^]**	1.9(1)	2.21(7)	1.61(7)	0.96(6)	0.33(7)
**z**	0.1671(2)	0.1672(1)	0.1669(2)	0.1672(2)	0.1658(3)
**B_4_** *6h* ***n*** **_Mn_**	0.09(1)	0.090(8)	0.093(8)	0.19(1)	0.12(1)
***n*** **_Al_**	0.08(2)	0.07(1)	0.07(1)	-	-
**B_iso_[Å^2^]**	1.8(1)	2.20(8)	1.68(8)	1.32(8)	0.9(1)
**x**	0.170(1)	0.168(1)	0.168(1)	0.168(1)	0.167(2)
**y**	0.341(3)	0.337(2)	0.337(2)	0.336(2)	0.333(4)
**B_5_** *12k* ***n*** **_Al_**	0.05(1)	0.030(7)	0.034(8)	0.076(7)	0.053(8)
**B_iso_[Å^2^]**	1.69(7)	1.77(4)	1.24(4)	0.73(4)	0.60(5)
**x**	0.1666(8)	0.1661(6)	0.1666(7)	0.1670(8)	0.170(2)
**y**	0.333(2)	0.332(1)	0.333(1)	0.334(2)	0.341(2)
**z**	0.08617(9)	0.08569(6)	0.08511(6)	0.08434(7)	0.0841(1)
** $\chi^{2}$ **	4.35	3.57	2.60	3.29	2.45

**Table 5:** EDX data collected for the selected intermetallic samples.

Sample	Atomic Percentage (at.%)						Apparent Composition (All Phases)
	La	Y	Ni	Mn	Al	O	
Y_1.00_	11.1(1)	12.2(2)	65.6(5)	3.6(1)	2.4(1)	5.1(6)	La_0.95_Y_1.05_Ni_5.63_Mn_0.31_Al_0.21_
Y_1.00_ Overview	10.6(1)	9.1(2)	66.4(5)	3.2(1)	2.1(2)	8.6(7)	La_1.08_Y_0.92_Ni_6.73_Mn_0.33_Al_0.21_
Y_1.33_	7.84(9)	17.3(2)	59.9(4)	4.1(1)	2.5(1)	8.4(6)	La_0.62_Y_1.38_Ni_4.77_Mn_0.33_Al_0.20_
Y_1.33_ Overview	7.6(1)	11.4(2)	67.5(5)	4.(1)	1.9(1)	7.6(6)	La_0.80_Y_1.20_Ni_7.09_Mn_0.43_Al_0.20_
Y_1.67_	3.49(6)	17.7(2)	66.3(4)	3.35(8)	2.1(1)	7.0(5)	La_0.33_Y_1.67_Ni_6.24_Mn_0.32_Al_0.20_
Y_1.67_ Overview	3.22(2)	15.3(2)	68.2(5)	3.3(1)	2.0(1)	7.9(6)	La_0.35_Y_1.65_Ni_7.38_Mn_0.36_Al_0.22_

**Table 6:** The selected sample nominal, chemical and refined compositions.

Sample	Nominal Composition	Average Chemical Composition from EDX (All Phases)	Refined Composition of the *H*-A_2_B_7_ Phase
Y_1.00_	LaYNi_6.5_Mn_0.33_Al_0.17_	La_1.01_Y_0.99_Ni_6.18_Mn_0.32_Al_0.21_	La_1.04_Y_0.96_Ni_6.47_Mn_0.33_Al_0.20_
Y_1.33_	La_0.67_Y_1.33_Ni_6.5_Mn_0.33_Al_0.17_	La_0.71_Y_1.29_Ni_5.93_Mn_0.38_Al_0.20_	La_0.70_Y_1.30_Ni_6.46_Mn_0.32_Al_0.23_
Y_1.67_	La_0.33_Y_1.67_Ni_6.5_Mn_0.33_Al_0.17_	La_0.34_Y_1.66_Ni_6.81_Mn_0.34_Al_0.21_	La_0.23_Y_1.77_Ni_6.50_Mn_0.34_Al_0.16_

**Table 7:** The obrained values of enthalpy and entropy of hydrogenation, and the calculated plateau pressure for the studied compositions, at 30 °C.

Plateau	Calculated P_eq_(bar) at 30 °C	ΔH_f_(kJ/mol)	ΔS_f_(J/(mol·K))	R^2^
Y_1.00_	1.45	−28.0	95.2	0.9913
Y_1.33_ lower plateau	0.08	−36.0	97.1	0.9894
Y_1.33_ higher plateau	0.26	−31.5	92.7	0.9454
Y_1.67_ lower plateau	0.14	−36.6	105.6	0.9954
Y_1.67_ higher plateau	0.75	−30.4	98.5	0.9991

**Table 8:** The maximum hydrogen storage capacity for the studied materials at different temperatures.

	50 °C	70 °C	90 °C
Y_1.00_	1.37 wt.%	1.32 wt.%	1.22 wt.%
Y_1.33_	1.57 wt.%	1.55 wt.%	1.49 wt.%
Y_1.67_	1.60 wt.%	1.57 wt.%	1.52 wt.%

## Data Availability

Data can be provided for reasonable requests.

## Supplementary Materials

The following supporting information can be downloaded at: https://www.mdpi.com/article/10.3390/molecules28093749/s1, Figure S1: In-situ data for all samples: (**a**) Y_0.67_, (**b**) Y_1.00_, (**c**) Y_1.33_, (**d**) Y_1.67_ The black lines pristine powder, the red lines after exposure to hydrogen; Figure S2: HR SR-PXD data collected for hydrogenated materials at MCX at Elettra Sinchrotron (Trieste). The samples were stored at ambient conditions in a sealed steel container with Ar gas and in a glovebox over a couple of months. The data were obtained under the same conditions as the SR-HR PXD data presented in Figure 1; Figure S3: SEM micrographs obtained for Y_1.00_ before hydrogen exposure (**a**), after hydrogen exposure (**b**) Y_1.33_ before hydrogen exposure (**c**), after hydrogen exposure (**d**) and Y_1.67_ before hydrogen exposure (**e**), after hydrogen exposure (**f**) at 25,000×; Figure S4: The chosen areas of collecting EDX data. (**a**) Y_1.00_, (**b**) Y_1.00_ overview. (**c**) Y_1.33_, (**d**) Y_1.33_ overview. (**e**) Y_1.67_, (**f**) Y_1.67_ overview; Figure S5: Example of kinetic curve of absorption relative to one point of the PCT measurement. The curve is obtained for a single point at 50 °C for sample Y_1.00_. At 1.5 h, the measured pressure was 3% lower (2.61 bar) than the pressure after 30 min absorption (2.69 bars).

## Abbreviations

The following abbreviations are used in this manuscript:

RE	Rare Earh
HIA	Hydrogen-Induced Amorphisation
HR SR-PXD	High-Resolution Synchrotron Radiation Powder X-ray Diffraction
PXD	Powder X-ray Diffraction
PND	Neutron Powder Diffraction
SEM	Scanning Electron Microscopy
EDX	Energy Dispersive X-ray Analysis
PCT	Pressure-Composition-Temperature
