# Supplementary material for: The Effect of Y Content on Structural and Sorption Properties of A2B7-Type Phase in the La–Y–Ni–Al–Mn System
Source: Molecules. 2023 Apr 27;28(9):3749. doi: 10.3390/molecules28093749 (PMC10180221; doi:10.3390/molecules28093749)
Supplement: Supplementary file 1 [file molecules-28-03749-s001.zip › molecules-2243573-supplementary.pdf]

Supplementary Materials: The effect of Y content on structural and sorption properties of A_2B_7-type phase in the La–Y–Ni–Al–Mn system

Emil H. Jensen [1]*, Loris Lombardo [2], Alessandro Girella [3], Matylda N. Guzik [1], Andreas Züttel [4], Chiara Milanese [3], Pamela Whitfield [5], Dag Noréus[6] and Sabrina Sartori [1]

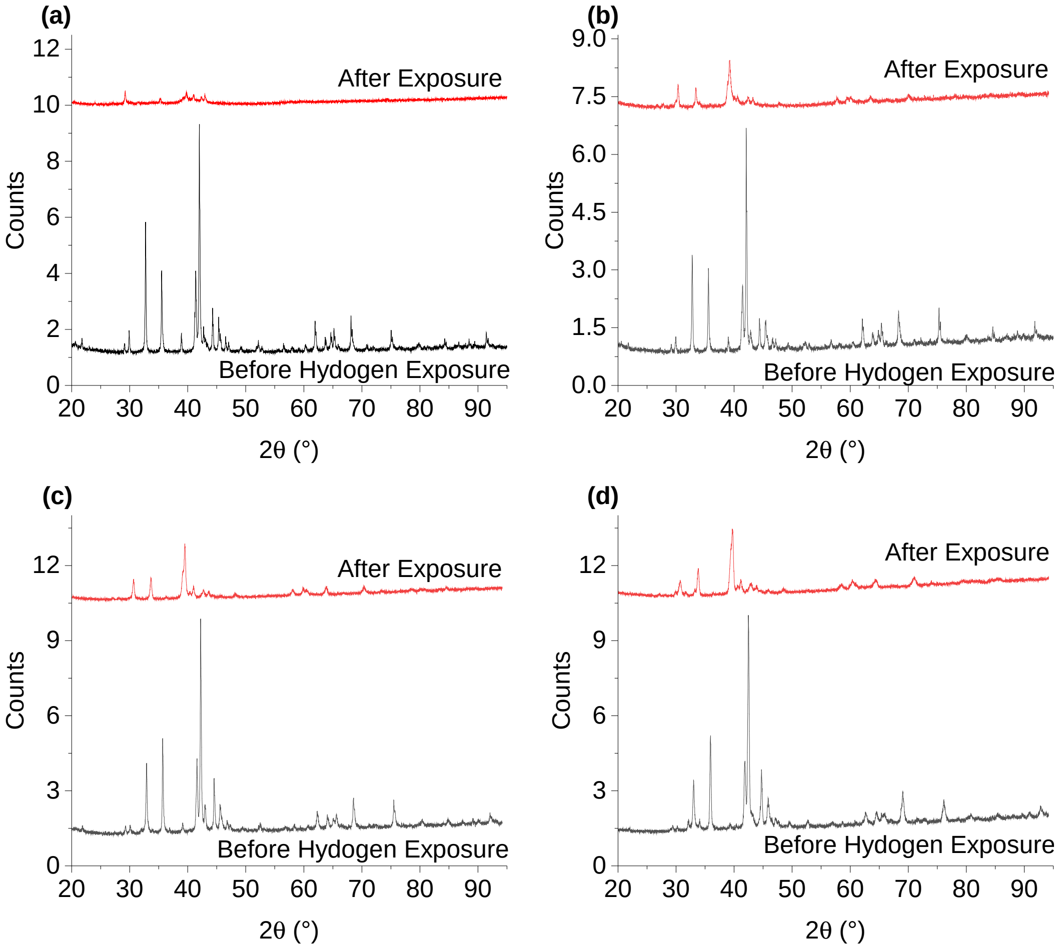

Figure S1. In-situ data for all samples: (*a*) $Y_{0.67}$, (*b*) $Y_{1.00}$, (*c*) $Y_{1.33}$, (*d*) $Y_{1.67}$ The black lines pristine powder, the red lines after exposure to hydrogen

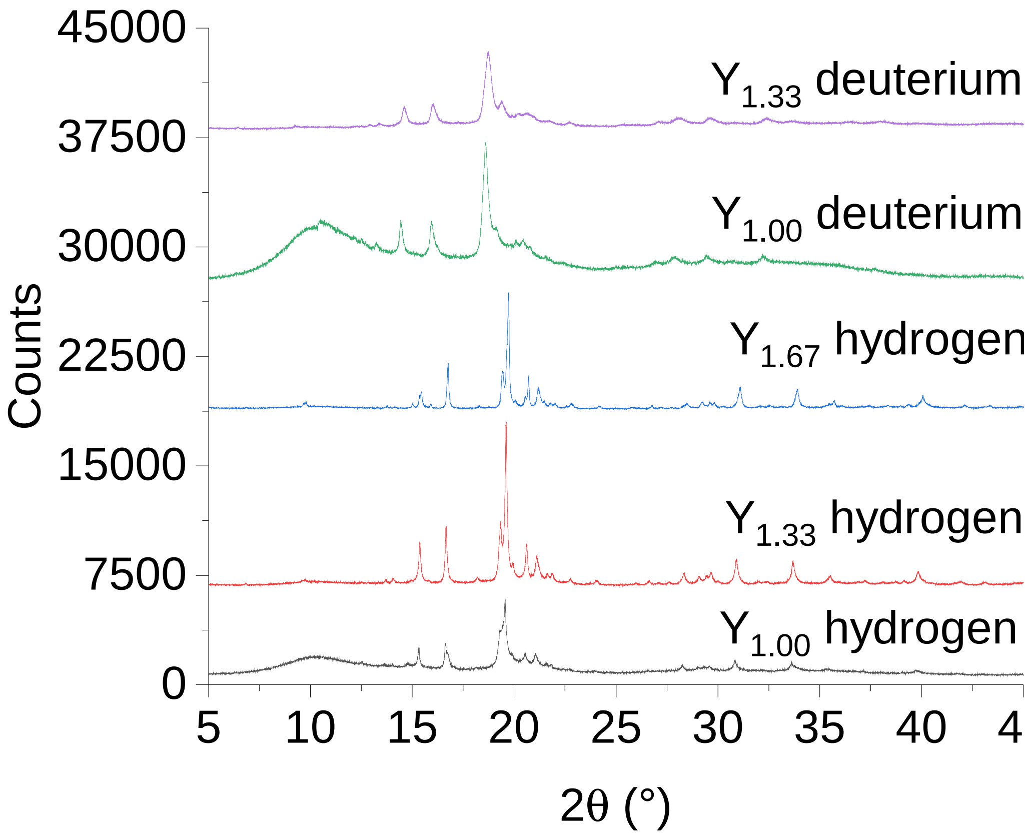

Figure S2. HR SR-PXD data collected for hydrogenated materials at MCX at Elettra Sinchrotron (Trieste). The samples were stored at ambient conditions in a sealed steel container with Ar gas and in a glovebox over a couple of months. The data were obtained under the same conditions as the SR-HR PXD data presented in Figure 1.

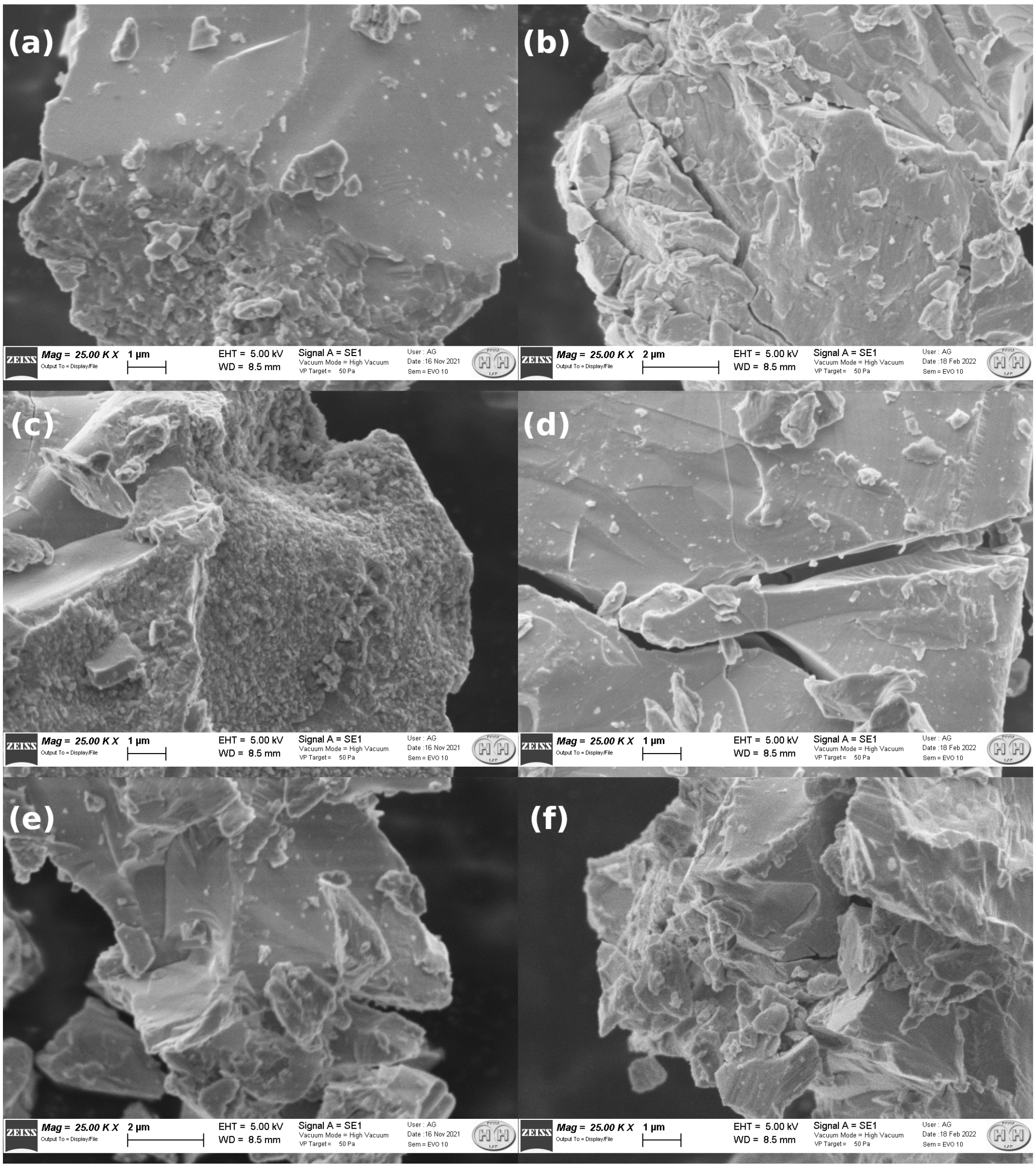

Figure S3. SEM micrographs obtained for $Y_{1.00}$ before hydrogen exposure (***a***), after hydrogen exposure (***b***) $Y_{1.33}$ before hydrogen exposure (***c***), after hydrogen exposure (***d***) and $Y_{1.67}$ before hydrogen exposure (***e***), after hydrogen exposure (***f***) at 25,000x

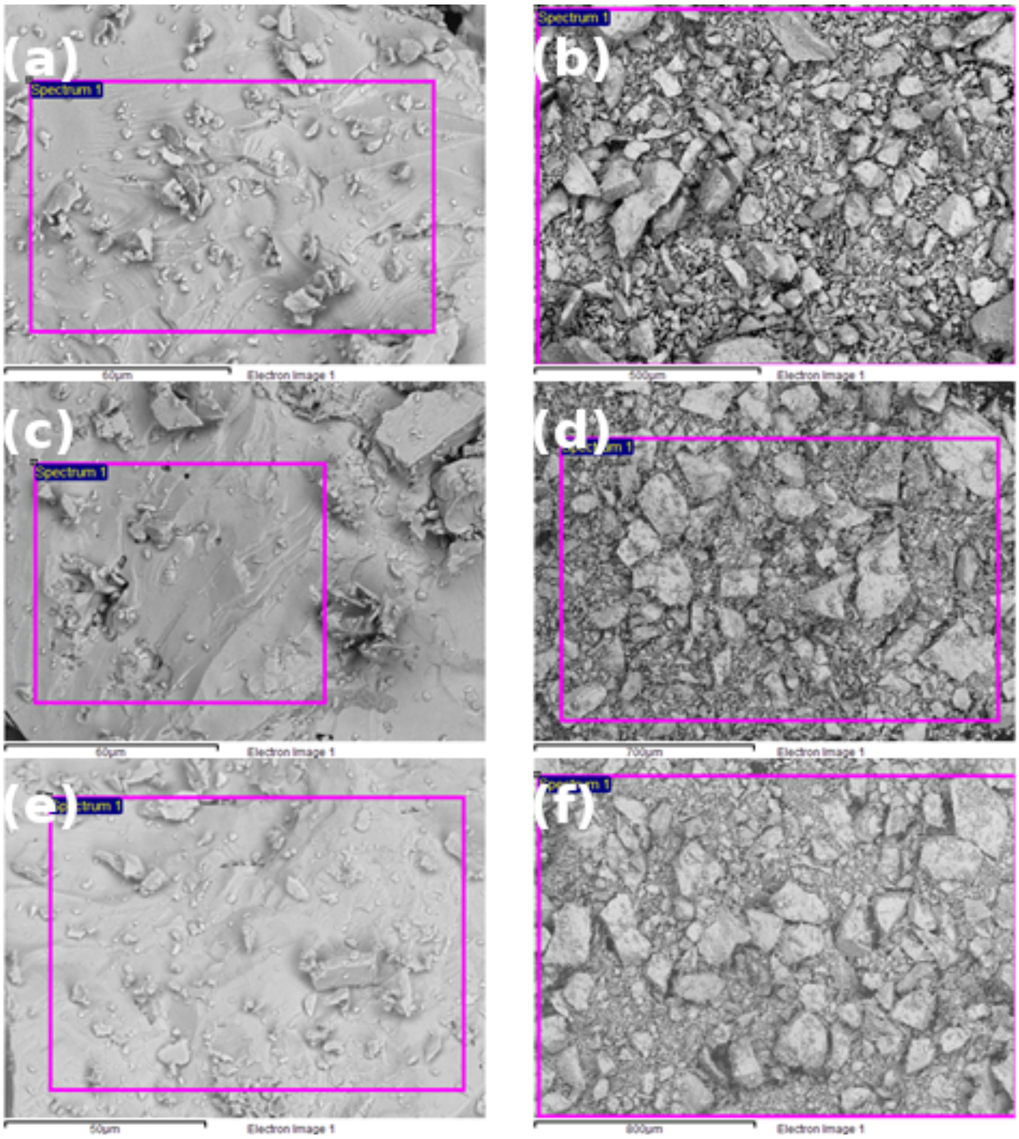

Figure S4. The chosen areas of collecting EDX data. (***a***) $Y_{1.00}$, (***b***) $Y_{1.00}$ overview. (***c***) $Y_{1.33}$, (***d***) $Y_{1.33}$ overview. (***e***) $Y_{1.67}$, (***f***) $Y_{1.67}$ overview

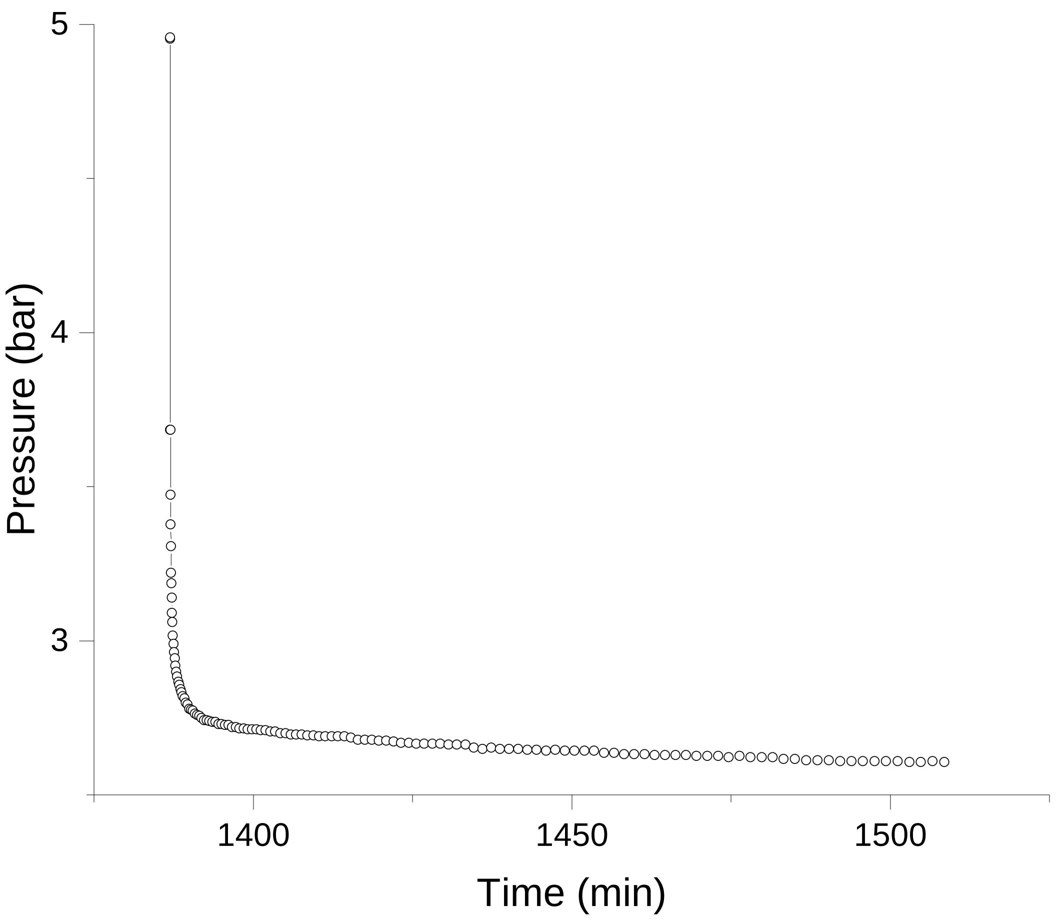

Figure S5. Example of kinetic curve of absorption relative to one point of the PCT measurement. The curve is obtained for a single point at 50°C for sample $Y_{1.00}$. At 1.5 hours, the measured pressure was 3% lower (2.61 bar) than the pressure after 30 min absorption (2.69 bars).
